# Supplementary material for: Venomix: a simple bioinformatic pipeline for identifying and characterizing toxin gene candidates from transcriptomic data
Source: PeerJ. 2018 Jul 31;6:e5361. doi: 10.7717/peerj.5361 (PMC6074769; doi:10.7717/peerj.5361)
Supplement: Supplemental Information 6 [file peerj-06-5361-s006.gz › FinalOutput_1E-6/Hyaluronidase_conohyal-Cn1_1/finaltree.pdf]

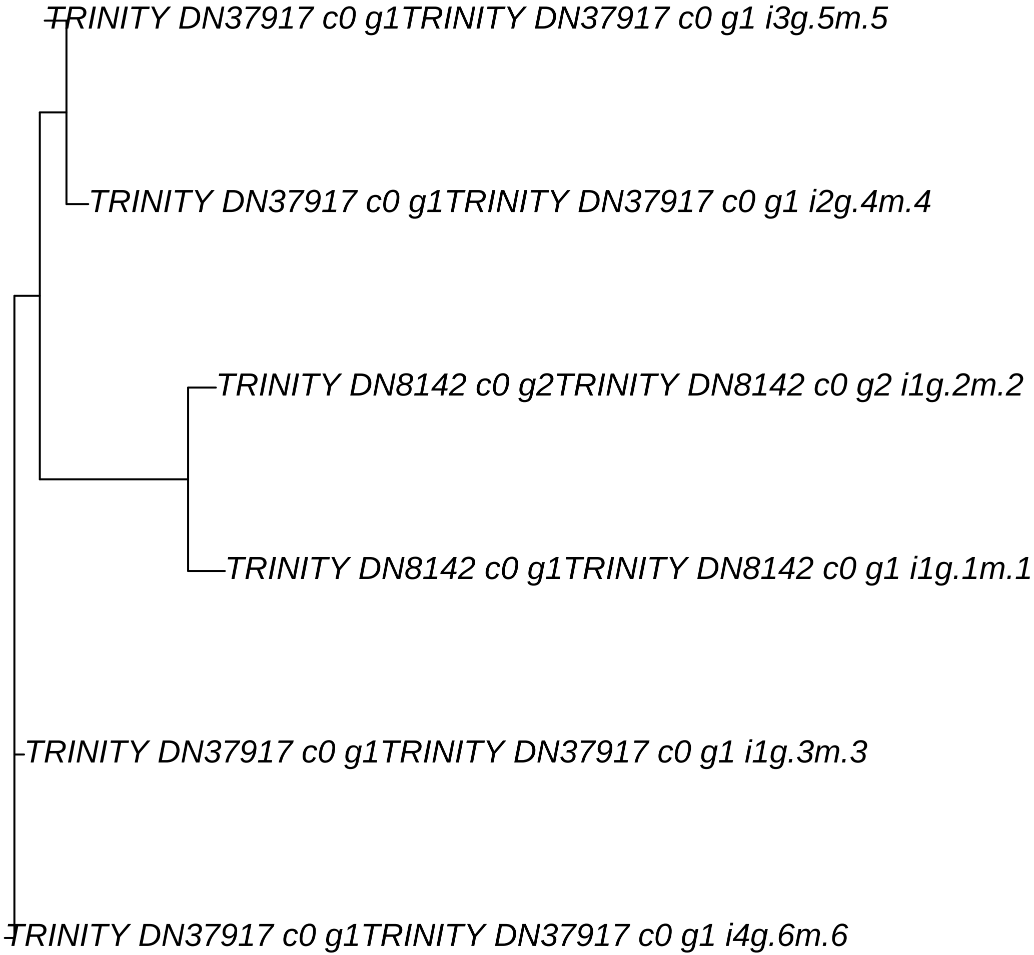
TRINITY DN37917 c0 g1TRINITY DN37917 c0 g1 i3g.5m.5
TRINITY DN37917 c0 g1TRINITY DN37917 c0 g1 i2g.4m.4
TRINITY DN8142 c0 g2TRINITY DN8142 c0 g2 i1g.2m.2
TRINITY DN8142 c0 g1TRINITY DN8142 c0 g1 i1g.1m.1
TRINITY DN37917 c0 g1TRINITY DN37917 c0 g1 i1g.3m.3
TRINITY DN37917 c0 g1TRINITY DN37917 c0 g1 i4g.6m.6
